# Craniofacial Mature Teratoma: A Case Report

**DOI:** 10.7759/cureus.104952

**Published:** 2026-03-09

**Authors:** Ahmed M Salloum, Amira Aladab, Sarah Arafat, Alyaa AlMadani, Ahmed AlGhafri

**Affiliations:** 1 Oral and Maxillofacial Surgery, Al Qassimi Hospital, Sharjah, ARE; 2 Plastic and Reconstructive Surgery, Al Qassimi Hospital, Sharjah, ARE; 3 Oral and Maxillofacial Surgery, Delta University for Science and Technology, Mansoura, EGY

**Keywords:** cleft palate, congenital craniofacial teratoma, epignathus, mature teratoma, skull base reconstruction

## Abstract

A congenital teratoma is a rare malformation. In this case report, we present a case of a one-month-old female patient with a craniofacial teratoma that has an intracranial origin from the skull base located anterior to the sella turcica, extending to the hard palate and associated with cleft palate. The patient presented to our department with a large tumor protruding from the oral cavity that did not cause an immediate respiratory obstruction, but there were feeding difficulties noted. The diagnosis was established based on clinical and radiological aspects and confirmed by a preoperative incisional biopsy. Tumor resection was performed, during which a barrier was created between the intracranial and extracranial spaces with calvarial bone graft and abdominal fat. The patient subsequently underwent a series of surgical excisions, reconstruction, and cleft palate repair. Histopathological examination revealed the typical components of a teratoma, including mature neuroglial tissue. After five years of follow-up, no regrowth was observed, and the patient had normal swallowing function without neurological deficits.

## Introduction

A teratoma is defined as a tumor consisting of multiple tissues that are not indigenous to their site of origin [1]. It is considered a true neoplasm of presumed primordial germ cell origin and typically consists of tissues derived from all three embryonic germ layers: ectoderm, mesoderm, and endoderm [2]. 

A teratoma can develop in almost any area of the body, but it is most commonly found in the midline. The reported incidence is approximately 1:4000 live births, with around 2%-9% occurring in the head and neck region. The most common sites are the sacrococcyx, anterior mediastinum, testis, ovary, and retroperitoneum. The term “epignathus” is used to describe a congenital teratoma arising from the oropharyngeal region [2]. 

Most of the reported cases in the literature result in stillbirth due to airway obstruction. Only a limited number of patients who underwent surgery in the neonatal period had a good outcome, and all survived [3]. In this report, we describe a rare case of a craniofacial teratoma with both intracranial and extracranial components in a female infant, associated with cleft palate.

## Case presentation

**Figure 1:**
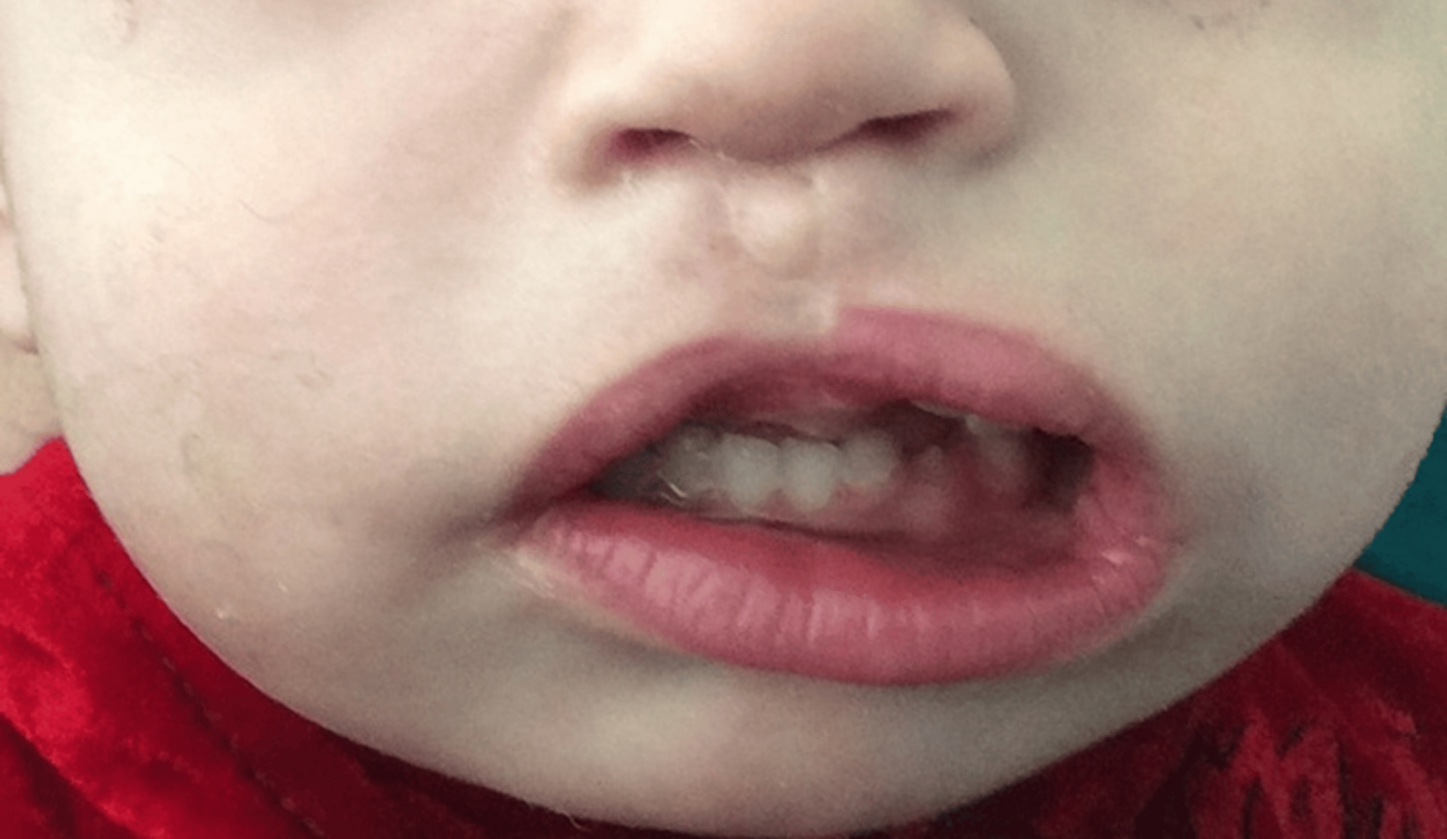
Mass in the premaxilla involving the upper anterior intraoral vestibule and the upper lip, measuring 2.5 × 2.5 cm, with concomitant cleft palate.

A one-month-old female patient was admitted to the Department of Maxillofacial and Plastic Surgery at Alexandria University Hospital with a mass in the premaxilla involving the upper anterior intraoral vestibule and the upper lip, measuring 2.5 × 2.5 cm, associated with a concomitant cleft palate (Figure 1). The remainder of the systemic examination was unremarkable, and no neurological deficits were detected. Due to difficulty with feeding, a nasogastric tube was inserted after delivery. The patient was born full-term to a 30-year-old multigravida mother via cesarean section following an uneventful pregnancy.

**Figure 2:**
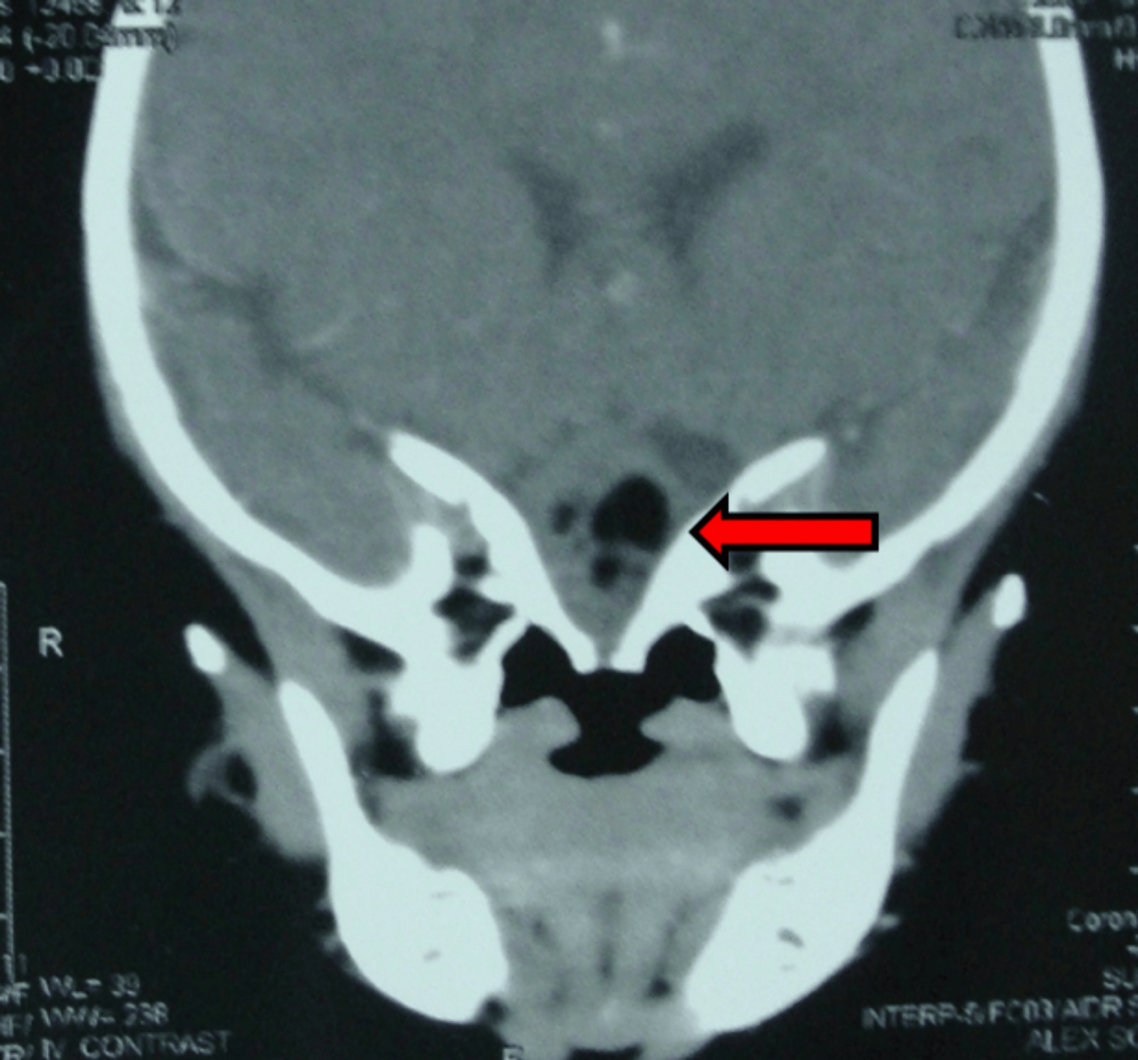
Axial CT scan showing a heterogeneous fat-density mass (red arrow) at the roof of the oral cavity, measuring 2.7 × 2.6 × 2.3 cm, with internal calcifications, communicating through an anterior skull base defect with an intracranial component of similar attenuation. CT: computerized tomography

Computed tomography (CT) revealed a heterogeneous mass predominantly containing fat density located at the roof of the oral cavity, measuring 2.7 × 2.6 × 2.3 cm in its maximum dimensions (Figure 2). Foci of calcification were identified within the lesion. The mass was seen in continuity with an osseous defect in the anterior skull base and demonstrated an intracranial component exhibiting a similar attenuation pattern, measuring 17 × 10 × 15 mm.

**Figure 3:**
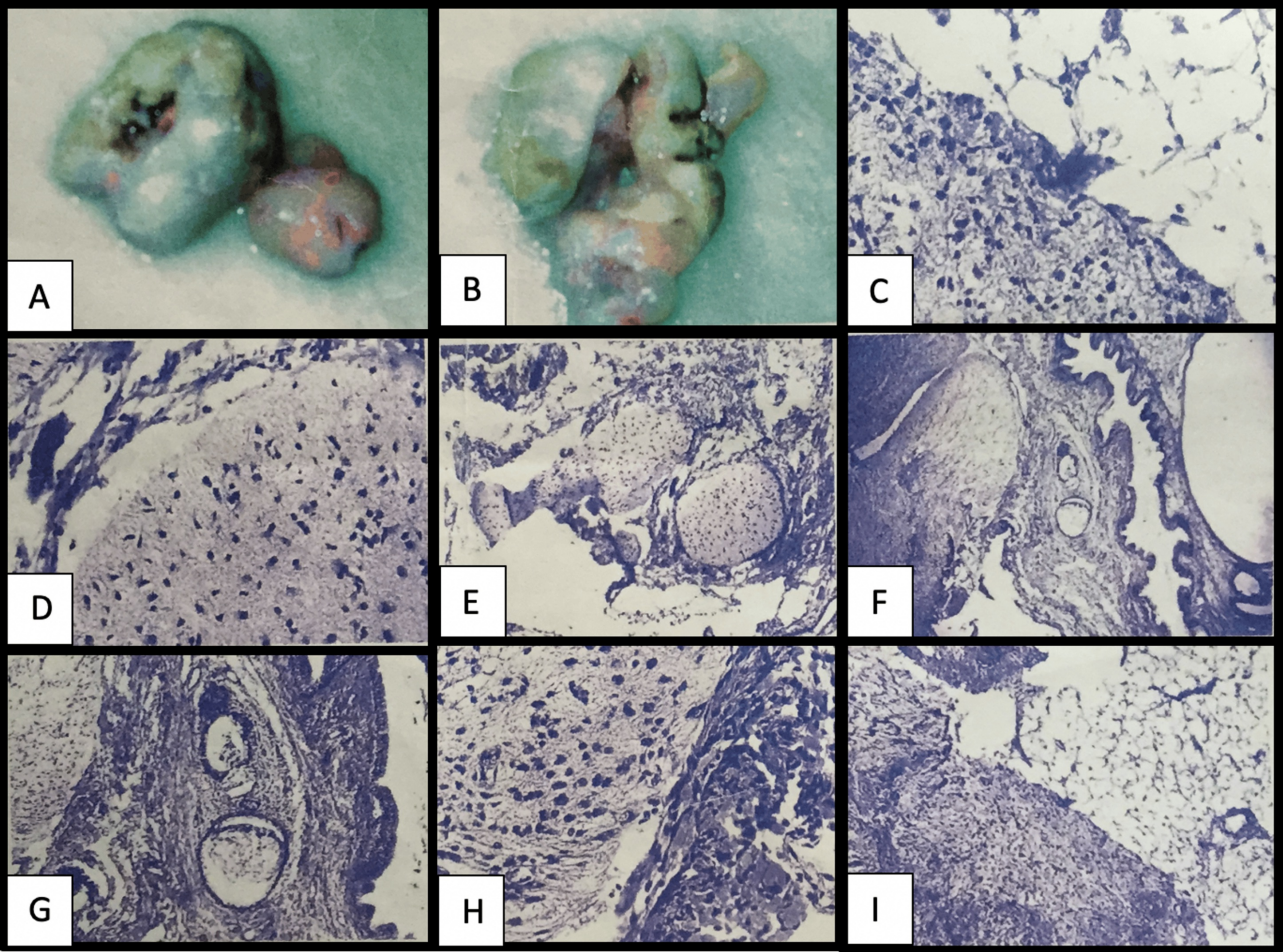
(A, B) Gross examination showed a soft tissue mass measuring 5 x 4 cm with a pink-whitish cut surface. Immunostaining done using the BenchMark Ventana System revealed positive reaction for synaptophysin and GFAP; however, it was negative for PLAP and AFP in the neural tissue. (C) Microscopic examination revealed an immature teratoma with intact surface squamous epithelium and mucosa underlined by islands of glial tissue with scattered neurons. (D) Microscopic examination revealed surface squamous epithelium, glial tissue and cerebellar-like tissue surrounded by mature adipose tissue and non-neoplastic submucosal elements. (E) Microscopic examination revealed the glial component dominated by proliferating oligodendroglial cells. (F) Microscopic examination revealed underlying glandular cystic areas lined by tall columnar epithelium. (G) Microscopic examination revealed associated microcalcifications observed in the glial component dominated by proliferating oligodendroglial cells. (H) Microscopic examination revealed that the tumor was predominated by glial tissue with multiple islands of immature-appearing cells; however, mitotic activity was not prominent. (I) Microscopic examination revealed the tumor extended to the resection margin. GFAP: glial fibrillary acidic protein; PLAP: placental-like alkaline phosphatase; AFP: alpha-fetoprotein

An incisional biopsy was performed (Figure 3), which revealed an intact surface squamous epithelium with underlying glandular cystic structures lined by tall columnar epithelium. The lesion showed a predominance of glial tissue with focal areas composed of immature-appearing cells. Microcalcifications were also identified. Immunohistochemical analysis was subsequently performed using glial fibrillary acidic protein (GFAP) and cytokeratin (CK). The tumor cells showed strong positivity for GFAP, confirming the glial component, while CK highlighted the epithelial elements. No definitive immature or malignant components were identified on further evaluation, supporting the diagnosis of a mature teratoma rich in neuroglial tissue.

Based on the clinical, radiological, and histopathological findings, a serial excision protocol was planned.

At two months of age, the patient underwent debulking of the intraoral mass. Local flaps were used to close the surgical defect. This procedure was primarily performed to facilitate adequate feeding and improve oral function. During the operation, a secondary cleft palate was identified for the first time.

At 10 months of age, a lip revision procedure was performed to improve aesthetic and functional outcomes. At one year and six months of age, palatoplasty was performed to repair the secondary cleft palate.

**Figure 4:**
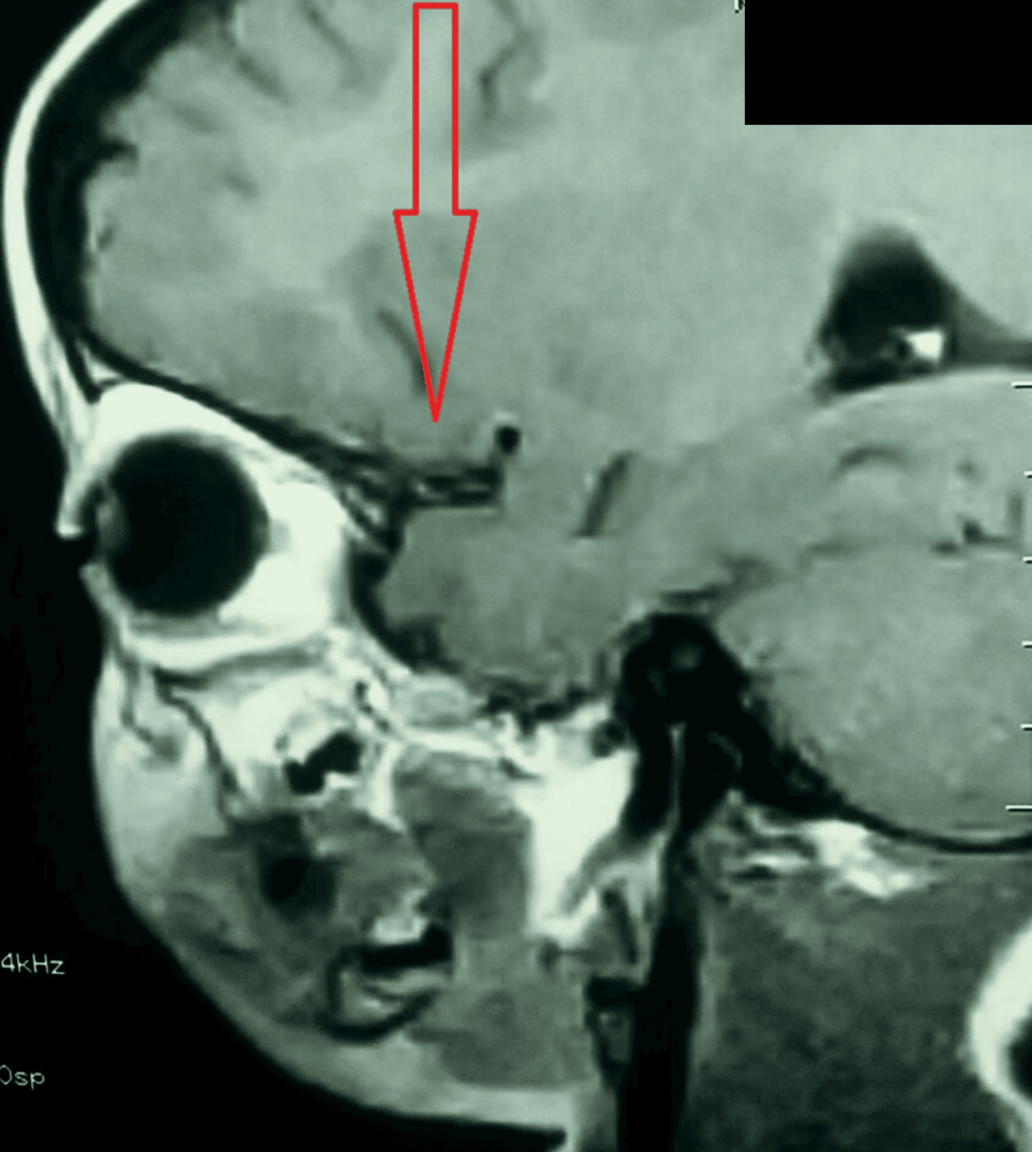
MRI demonstrated an intracranial component of the lesion located anterior to the sella turcica, abutting the inferior surfaces of the bilateral frontal lobes. The lesion (red arrow) extended inferiorly through the anterior skull base into the submucosal region of the roof of the oral cavity. MRI: magnetic resonance imaging

At two years of age, follow-up magnetic resonance imaging (MRI) demonstrated persistence of the intracranial component located anterior to the sella turcica, abutting the inferior surfaces of the bilateral frontal lobes and extending inferiorly along the submucosal region of the roof of the oral cavity (Figure 4).

**Figure 5:**
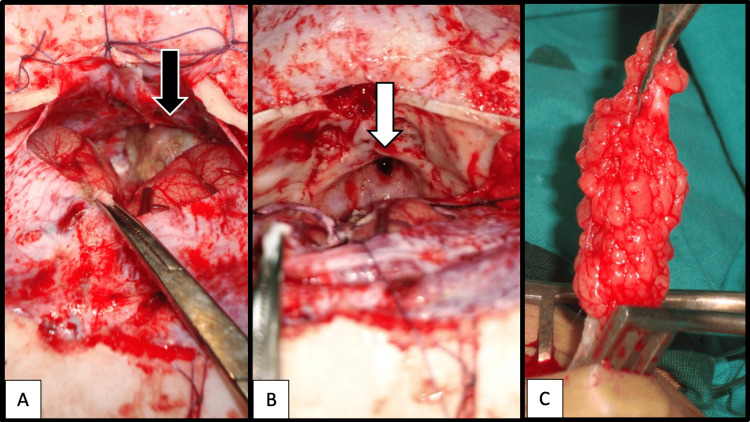
Intraoperative images showing the surgical site: (A) black arrow indicates the tumor, (B) white arrow highlights the hollow defect after complete intracranial excision via a transcranial approach, and (C) reconstruction using abdominal fat graft.

**Figure 6:**
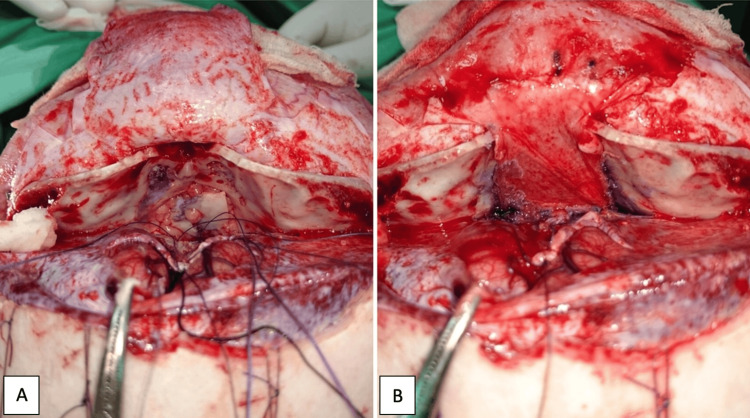
(A) Intraoperative view showing reconstruction of the skull base using a calvarial bone graft and abdominal fat graft, secured with Histoacryl. (B) Dural repair using a pericranial flap, achieving a complete seal between the intracranial and extracranial compartments.

Subsequently, at two years and four months of age, complete intracranial excision of the tumor (Figure 5A) was performed via a transcranial approach. Following tumor resection (Figure 5B), reconstruction was then completed using abdominal fat (Figure 5C) and a calvarial bone graft to seal the communication between the intracranial and extracranial compartments (Figure 6A). Dural repair was achieved using a pericranial flap by the neurosurgical team (Figure 6B).

**Figure 7:**
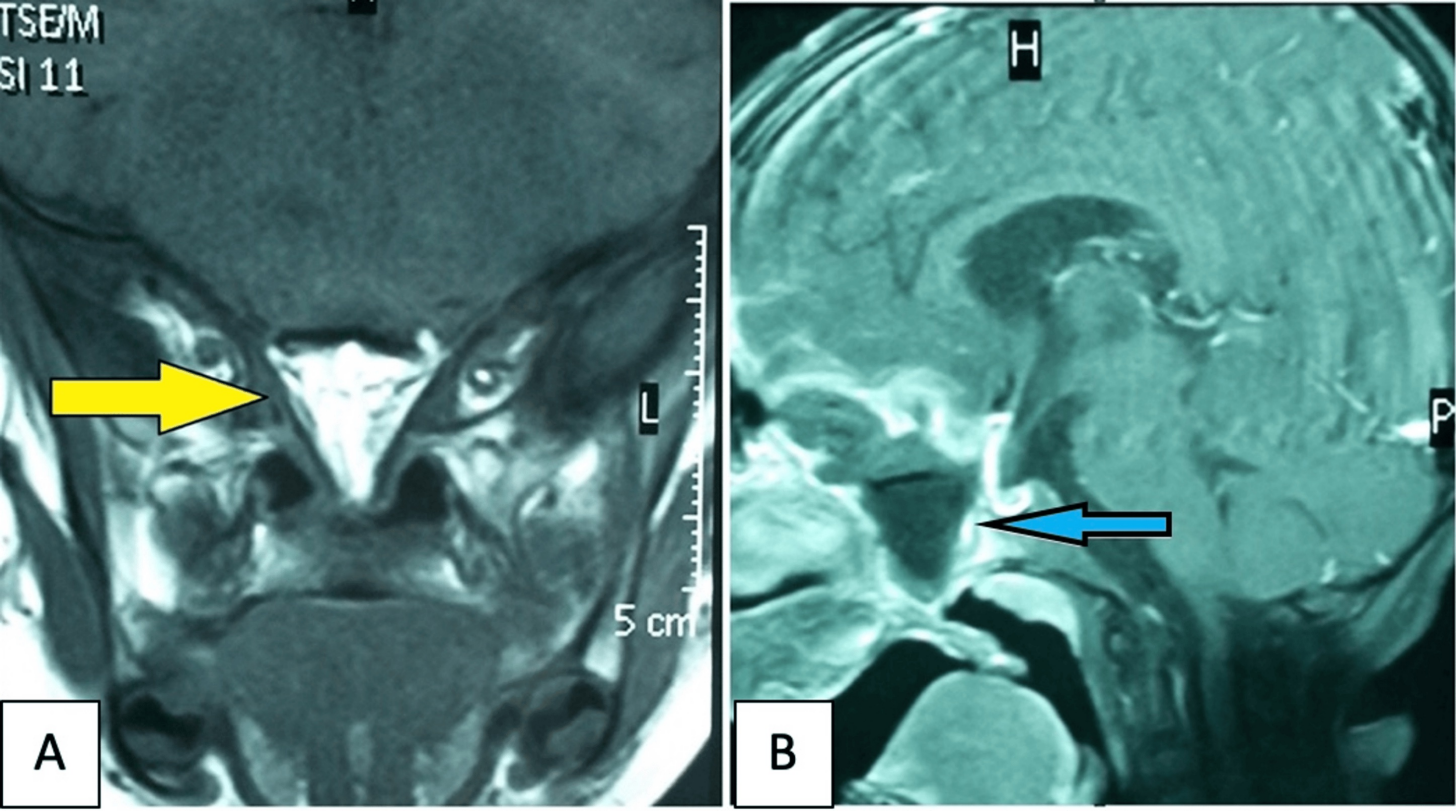
Postoperative MRI of both coronal (A) and sagittal (B) views showing complete resection of the immature teratoma and successful separation of the intracranial and intraoral compartments. Arrows indicate the T1-hypointense (blue arrow) and T2-hyperintense (yellow arrow) regions corresponding to the surgical site. MRI: magnetic resonance imaging

Three months after the fourth and final surgery, follow-up MRI demonstrated no evidence of tumor recurrence (Figure 7). At four years of age, the patient remained healthy, with normal growth and age-appropriate cognitive development.

## Discussion

A mature teratoma is a neoplasm that develops during intrauterine life. Although it is a rare neoplasm, it presents in about 2.5% of all neoplasms affecting the pediatric population [4]. According to Solis-Pazmino et al., cleft palate is the most common anomaly associated with teratomas [5], as observed in our case.

Neonatal teratomas may present with both intracranial and extracranial components, a presentation that is rarely reported in the literature [6]. In our patient, the tumor was primarily intracranial, extending into the maxillary region. Consequently, the surgical approach prioritized achieving optimal functional outcomes, including breathing, feeding, and speech, over immediate complete excision.

Teratomas are classified into three subtypes: mature, immature, and malignant. Mature teratomas are characterized by well-organized, differentiated tissues and are the most commonly diagnosed type in infancy [5]. The etiology of teratomas remains uncertain. Some hypotheses suggest that the tumor arises from totipotent cells sequestered during embryogenesis [2], while others propose that epignathus develops from pluripotent cells in Rathke’s pouch, leading to disorganized tissue growth [7,8]. Moreover, the mortality rate associated with palatal teratomas has decreased in recent years due to advances in antenatal imaging, which allow early diagnosis and enable multidisciplinary teams to plan appropriate postnatal surgical interventions [7,8].

Management strategies vary according to the tumor size and clinical presentation. Large obstructive tumors often require emergency intervention to secure the airway, whereas small, nonobstructive lesions may be monitored initially. Patients with epignathi are particularly at risk of airway compromise and high mortality. Surgical intervention aims for complete resection whenever feasible and may be performed in stages when both intracranial and extracranial components are present. Associated deformities, such as cleft palate, velopharyngeal insufficiency, and facial asymmetry, can be addressed in subsequent stages to optimize functional outcomes related to feeding, speech, and appearance [8].

## Conclusions

Skull base reconstruction is essential in the surgical management of intracranial teratomas that perforate the cranial base, which coincides with our protocol. In our case, treatment was tailored across four staged operations, with skull base reconstruction achieved using a calvarial bone graft and abdominal fat graft. Neonatal mature teratomas with combined intracranial and extracranial extension are rare and pose significant diagnostic and therapeutic challenges. Early antenatal detection and meticulous multidisciplinary planning are critical for optimizing survival and functional outcomes. Moreover, in cases where complete excision carries a high risk of morbidity, prioritizing airway protection and preservation of essential functions such as breathing, feeding, and speech is justified. Staged surgical management enables safe tumor control, effective skull base reconstruction, and subsequent correction of associated anomalies such as cleft palate. This case report highlights the importance of an individualized, function-oriented treatment strategy in managing complex epignathus teratomas involving the cranial base.
